# Vitamin D and Cognitive Impairment

**DOI:** 10.3390/nu17081301

**Published:** 2025-04-09

**Authors:** Nalinee Imerbsin, Prapimporn Chattranukulchai Shantavasinkul, Pirada Witoonpanich, Jintana Sirivarasai, Naphat Taonam, Pariya Phanachet, Daruneewan Warodomwichit, Kulapong Jayanama, Kochawan Boonyawat, Nicha Somlaw, Boonsong Ongphiphadhanakul, Daochompu Nakawiro, Sookjaroen Tangwongchai

**Affiliations:** 1 Doctor of Philosophy Program in Nutrition, Faculty of Medicine Ramathibodi Hospital and Institute of Nutrition, Mahidol University, Bangkok 10400, Thailand; nalinee.ime@mahidol.edu; 2 Department of Pharmacy, Faculty of Medicine Ramathibodi Hospital, Mahidol University, Bangkok 10400, Thailand; 3 Division of Nutrition and Biochemical Medicine, Department of Medicine, Faculty of Medicine Ramathibodi Hospital, Mahidol University, Bangkok 10400, Thailand; nutrinaphatta@gmail.com (N.T.); jphanachet@yahoo.com (P.P.); daruneewanw@gmail.com (D.W.); 4 Nutrition Unit, Faculty of Medicine Ramathibodi Hospital, Mahidol University, Bangkok 10400, Thailand; jintana.sir@mahidol.ac.th; 5 Division of Neurology, Department of Medicine, Faculty of Medicine Ramathibodi Hospital, Mahidol University, Bangkok 10400, Thailand; piradaw@gmail.com; 6 Department of Medicine, Chakri Naruebodindra Medical Institute, Faculty of Medicine Ramathibodi Hospital, Mahidol University, Samut Prakan 10400, Thailand; kulapong.jay@mahidol.ac.th; 7 Division of Hematology, Department of Medicine, Faculty of Medicine Ramathibodi Hospital, Mahidol University, Bangkok 10400, Thailand; kochawan.boo@mahidol.ac.th; 8 Division of Clinical Nutrition, Department of Medicine, Faculty of Medicine, King Chulalongkorn Memorial Hospital, Thai Red Cross Society, Chulalongkorn University, Bangkok 10330, Thailand; dr.nichasomlaw@gmail.com; 9 Division of Endocrinology and Metabolism, Faculty of Medicine Ramathibodi Hospital, Mahidol University, Bangkok 10400, Thailand; boonsong.ong@mahidol.ac.th; 10 Department of Psychiatry, Faculty of Medicine Ramathibodi Hospital, Mahidol University, Bangkok 10400, Thailand; pinkstar1973@gmail.com; 11 Department of Psychiatry, Faculty of Medicine, Chulalongkorn University, Bangkok 10330, Thailand; 12 Center of Excellence in Cognitive Impairment and Dementia, Faculty of Medicine, Chulalongkorn University, Bangkok 10330, Thailand

**Keywords:** vitamin D, mild cognitive impairment, Alzheimer’s disease, adiposity, insulin resistance, older population

## Abstract

Background: Vitamin D deficiency is recognized as a significant public health concern, and it has been identified as one of the potentially modifiable risk factors for mild cognitive impairment (MCI). However, evidence regarding the relationship between vitamin D status and cognitive function remains conflicting. Objective: Therefore, this study aimed to examine the prevalence of vitamin D deficiency in the Thai elderly population and an association between vitamin D status and cognitive function, adiposity, and insulin sensitivity. Methods: This study enrolled participants aged 55–80 years with normal cognitive function (normal group) or MCI from the prospective cohort in the “Holistic approach of Alzheimer’s disease in Thai people (HADThai study)”. We used the baseline clinical data to determine the prevalence of vitamin D deficiency and its association between vitamin D status and cognitive function, adiposity, and insulin sensitivity. Results: A total of 718 subjects (71.9% women) with a mean age of 65.7 ± 5.8 years and a mean BMI of 23.9 ± 3.7 kg/m^2^ were enrolled. There were 470 (65.5%) participants with normal cognitive function and 248 (34.5%) with MCI. Vitamin D status did not differ significantly between individuals with normal cognitive function and those with MCI. The prevalence of vitamin D deficiency (<20 ng/mL) and vitamin D inadequacy (<30 ng/mL) in both normal cognitive function and MCI was around 6.5% and 40.0%, respectively. While serum 25(OH)D concentrations were inversely associated with body mass index (BMI), body fat, %body fat, and the homeostasis model assessment of insulin resistance (HOMA-IR), no relationship was found between vitamin D status and cognitive function. Conclusions: Our study emphasized the high prevalence of vitamin D inadequacy among elderly individuals and an inverse association of vitamin D status and adiposity and insulin resistance. These findings emphasize the importance of addressing vitamin D deficiency in the elderly population to improve overall health outcomes. Nevertheless, our results do not support a direct role of vitamin D status in cognitive decline in this population. Further research, particularly studies with longer follow-up periods and the inclusion of patients with dementia with details of vitamin D supplementation, is needed to clarify the potential role of vitamin D in cognitive decline and neurodegenerative diseases.

## 1. Introduction

Vitamin D deficiency has emerged as a global health concern, affecting populations across various geographic regions. Surprisingly, even in Thailand, a tropical country located in Southeast Asia with abundant year-round sunlight, vitamin D deficiency remains prevalent, with reported rates ranging between 4.0% to 30.1% in the general population [1,2,3,4,5,6,7,8]. Interestingly, in Thailand, vitamin D deficiency appears to be more common among younger individuals. Moreover, the female sex and urbanization have been identified as key risk factors for this deficiency. While the precise mechanisms underlying this phenomenon remain unclear, it may be linked to cultural preferences for paler skin, which can lead to avoiding sun exposure and inadequate vitamin D synthesis. Additionally, obesity is a recognized risk factor for vitamin D inadequacy, potentially due to mechanisms such as the sequestration of vitamin D in adipose tissue, thereby reducing its bioavailability [7].

Elderly individuals are an at-risk group for developing vitamin D deficiency due to several factors such as reduced skin synthesis of vitamin D, lower dietary intake, and diminished sun exposure [2,4]. Although vitamin D is widely recognized for its crucial role in calcium and phosphorus regulation and its impact on bone health, growing evidence suggests it may also exert significant effects beyond the skeletal system [9,10,11]. Specifically, active vitamin D has been linked to brain health and cognitive function, with the presence of vitamin D receptors (VDRs) in brain cells suggesting a potential role in neuroprotection and the prevention of neurodegenerative diseases [12,13].

Mild cognitive impairment (MCI) represents an intermediate state between normal cognitive decline of aging and dementia, mostly in the form of Alzheimer’s disease. MCI may progress to dementia with an annual rate of progression of 5–17% [14,15,16]. The risk factors associated with cognitive decline and dementia include aging, genetic predisposition, and vascular risk factors such as diabetes, hypertension, dyslipidemia, and obesity. MCI is generally defined by the presence of memory loss greater than expected for age, objective cognitive impairment in one or more domain, but the preserved ability to function in daily activity [17]. The Montreal Cognitive Assessment (MoCA) is the recommended cognitive screening tool for detecting MCI [17,18]. The management of MCI is critical since some treatment modalities may slow diseases progression or even reverse the MCI to normal cognitive function.

Vitamin D deficiency has been identified as one of the potential modifiable risk factors for MCI and dementia [19]. Thus, the addressing and treatment of vitamin D deficiency, which may potentially slow or reverse cognitive decline, have become a focus of research. In vitro and animal studies have demonstrated that vitamin D has neuroprotective and anti-inflammatory effects and may reduce the accumulation of amyloid plaque, a hallmark of Alzheimer’s disease, in the brain [20]. Specifically, 1,25-dihydroxyvitamin D (1,25(OH)2D) has been shown to enhance the ability of macrophages to phagocytose amyloid-β protein [21] and reduce amyloid plaque deposition [22,23]. These findings suggest that vitamin D may play a role in mitigating neurodegenerative processes. However, human studies exploring the relationship between vitamin D status and cognitive function have yielded conflicting results [24,25,26,27,28,29,30,31,32,33,34,35,36].

Therefore, the primary objective of this study was to explore the association between vitamin D status and cognitive function, adiposity, and insulin sensitivity. By better understanding the potential links between vitamin D and these factors, we expect to contribute to the expanding body of research on modifiable risk factors for cognitive decline and metabolic health in aging populations. Understanding these relationships could inform targeted interventions to improve cognitive and metabolic outcomes in the elderly, particularly in regions like Thailand where vitamin D deficiency persists despite abundant sunlight. Additionally, the relationship between vitamin D deficiency, cognitive function, and metabolic health remains poorly understood, particularly in elderly populations in Southeast Asia. Our study bridges this gap by exploring these associations in a Thai elderly cohort, providing data that can inform preventive and therapeutic strategies.

## 2. Materials and Methods

### 2.1. Participants

This prospective cohort study entitled the “Holistic approach of Alzheimer’s disease in Thai people (HADThai study)” was conducted between June 2018 and March 2023 at 3 major medical institutions in Bangkok, Thailand: (1) Chulabhorn Hospital, Chulabhorn Royal Academy, (2) King Chulalongkorn Memorial Hospital, Thai Red Cross Society, and (3) Ramathibodi Hospital, Mahidol University. Participants aged 55 to 80 years with either normal cognitive function (normal group) or mild cognitive impairment (MCI group) were enrolled and followed up with every 3 months for one year. Baseline demographic data, including age, sex, education level, self-reported history of smoking, alcohol consumption, medical comorbidities, and vitamin supplementation, were collected. Cognitive function and body composition analyses were assessed at baseline, 6 months, and 12 months, while blood biochemistry measures were collected at baseline and 12 months. In this analysis, we used only baseline clinical data to assess the prevalence of vitamin D deficiency and examine its association with cognitive function, adiposity, and insulin sensitivity.

The exclusion criteria included participants with a history of neurological disorders (ischemic or hemorrhagic stroke, seizures, Parkinson’s disease, and amyotrophic lateral sclerosis (ALS)), depression, advanced kidney disease (defined as an estimated glomerular filtration rate (eGFR) < 30 mL/min/1.73 m^2^), cirrhosis (Child–Pugh class C), poorly controlled diabetes (HbA1C > 7%), a history of malignancy, HIV infection, syphilis, and deficiencies in vitamin B12 or folate. All participants underwent brain magnetic resonance imaging (MRI), and those with evidence of vascular dementia, grade III or IV white matter lesions, focal brain lesions, intra- or extracranial masses, or signs of brain infarction or hemorrhage were excluded from the study.

The study was reviewed and approved by the Human Research Ethics Committees of Chulabhorn Hospital, King Chulalongkorn Memorial Hospital, and the Faculty of Medicine, Ramathibodi Hospital. Detailed explanations of the study protocol were provided to all participants and/or their caregivers who were given the opportunity to ask questions. Written informed consent was obtained from all participants or their caregivers prior to enrollment, and the study was conducted in accordance with the principles outlined in the Declaration of Helsinki.

### 2.2. Cognitive Function Test and Diagnosis of MCI

Cognitive function was assessed using the Montreal Cognitive Assessment (MoCA) [37] or MoCA-B for those who had less than 4 years of education [38], Mini-Mental State Examination (MMSE) [39], and the Clinical Dementia Rating scale (CDR) [40,41]. Depression was assessed using the Thai version of the 30-item Geriatric Depression Scale (TGDS-30) [42]. Participants were classified as having either normal cognitive function or mild cognitive impairment (MCI) based on modified Peterson Criteria [43] conducted by clinical experts in dementia, including psychiatrists and neurologists. The diagnosis was determined through the clinical assessment of the participants and caregivers, as well as various cognitive tests. Subjects with normal cognition were required to have normal scores on the MMSE and MoCA/MoCA-B, a Global CDR score of 0, and no active depression with TGDS-30 scored less than 10, or have no other psychiatric disorders. For MCI subjects, normal scores on the MMSE were required, along with MoCA/MoCA-B scores below 25 and a CDR of 0.5. The MoCA scale was one of the screening tools for MCI. It assesses multiple domains of cognition, including memory, visuospatial and executive functions, naming, attention, abstraction, language, and orientation. MoCA scores range from 0 to 30, with higher scores reflecting better cognitive function. A cutoff score of less than 25 was used on the MoCA and MoCA-B to identify participants with MCI.

### 2.3. Vitamin D and Blood Chemistry Measurements

All blood samples were fractionated using a standard procedure and stored at −80 °C until analysis. Serum 25-hydroxyvitamin D [25(OH)D] concentrations, including 25(OH)D2 and 25(OH)D3, were measured by liquid chromatography coupled with tandem mass spectrometry (LC-MS/MS, Agilent 1260 Infinity liquid chromatograph, Agilent Technologies, Waldbronn, Germany) coupled to a QTRAP^®^ 5500 tandem mass spectrometer (AB SCIEX, Foster City, CA, USA). All analyte values of the calibrator (Chromsystems 3PLUS1^®^ Multilevel Serum Calibrator Set 25-OH-Vitamin D3/D2) and control (MassCheck^®^ 25-OH-Vitamin D3/D2) used in this study were traceable to certified substances and standard reference materials of the National Institute of Standards and Technology ([NIST] 972 and NIST 972a, respectively). The summation of serum 25(OH)D3 and 25(OH)D2 was used to reflect vitamin D status. The inter-assay and intra-assay coefficients of variation of total serum 25(OH)D levels were 7.2% and 5.3%, respectively. In addition, traceability tests using a certified standard reference material (SRM 972a) from NIST were also carried out and showed concordant results with the assigned values of NIST SRM 972a. Vitamin D status was categorized as follows: inadequacy (25(OH)D < 30 ng/mL), insufficiency (25(OH)D between 20 and 30 ng/mL), and deficiency (25(OH)D < 20 ng/mL) [6].

Fasting plasma glucose (FPG) was measured by photometric and potentiometric measurements (ALINITY C, Abbott, Chicago, IL, USA). Hemoglobin (Hb) A1C was measured by photometric transmission measurements (Tina-quant, Roche, Mannheim, Germany). Serum insulin levels were determined by chemiluminescent microparticle immunoassay (CMIA) technology (ALINITY I, Abbott, Wiesbaden, Germany). Insulin resistance was estimated using the homeostasis model assessment (HOMA-IR) based on FPG and insulin concentrations, calculated using the HOMA-2 model [44].

### 2.4. Body Composition Measurements

Anthropometric variables including weight, height, and waist circumference were measured using standard techniques. Body mass index (BMI) was derived by weight (kg)/height (m)^2^. Body composition was determined after at least 8 h of fasting using a multifrequency bioelectrical impedance analysis (BIA) with eight-point tactile electrodes (InBody 770; Biospace, Seoul, Republic of Korea). Body fat percentages (%BF) and skeletal muscle mass percentages (%SMM) were calculated by (body fat mass/body weight) × 100 and (SMM/body weight) × 100, respectively.

### 2.5. Statistical Analysis

Statistical analyses were performed using STATA 18.0 (StataCorp. 2023. Stata Statis-tical Software: Release 18, StataCorp LLC., College Station, TX, USA). Data were presented as mean and standard deviation (SD) for continuous variable and frequency (%) for binary and categorical variables. The continuous variables were compared using independent sample t-tests and by estimating the mean difference with a 95% confidence interval (CI). The test results of categorical variables were evaluated by a chi-square test or Fisher’s exact test as appropriate. The Pearson correlation was used to demonstrate the association of 25(OH)D status and variables. Univariable and multivariable logistic regression analyses were used to determine independent factors associated with cognitive function and the estimated odds ratio (OR) with a 95% CI. The results were deemed statistically significant at *p* < 0.05.

## 3. Results

A total of 718 participants were recruited in the study, with 516 (71.9%) being women. Of the participants, 470 (65.5%) had normal cognitive function, while 248 (34.5%) were diagnosed with MCI. The mean age ± SD was 65.7 ± 5.8 years, and the mean BMI ± SD was 23.9 ± 3.7 kg/m^2^. Participants with MCI were significantly older, completed fewer years of education, and had a higher prevalence of cardiovascular diseases compared to those with normal cognitive function. MMSE and MoCA scores were significantly lower in the participants with MCI. The clinical characteristics of the study population stratified by cognitive status are shown in Table 1.

The prevalence of vitamin D inadequacy (25(OH)D < 30 ng/mL), vitamin D insufficiency (20–30 ng/mL), and vitamin D deficiency (<20 ng/mL) in elderly Thai individuals was 40% (287 participants), 33.4% (240 participants), and 6.5% (47 participants), respectively. The mean vitamin D level across all participants was 34.5 ± 12.6 ng/mL, and vitamin D status was comparable between individuals with normal cognitive function and those with MCI. Approximately 199 participants (27.7%) were taking vitamin D supplements, with no significant difference in supplement usage between the cognitive groups.

Participants with MCI exhibited significantly higher BMI, waist circumferences, hip circumferences, and waist–hip ratios compared to those with normal cognitive function. Additionally, individuals with MCI had significantly higher levels of body fat, percentage of body fat, and visceral fat area. Skeletal muscle mass was not different between the groups; however, the percentage of skeletal muscle mass was slightly lower in participants with MCI (Table 2). Fasting plasma glucose and HbA1C levels were significantly higher in the MCI group. Although participants with mild cognitive impairment (MCI) had slightly higher levels of insulin and HOMA-IR compared to those with normal cognitive function, these differences did not reach statistical significance (Table 3).

**Figure 1:**
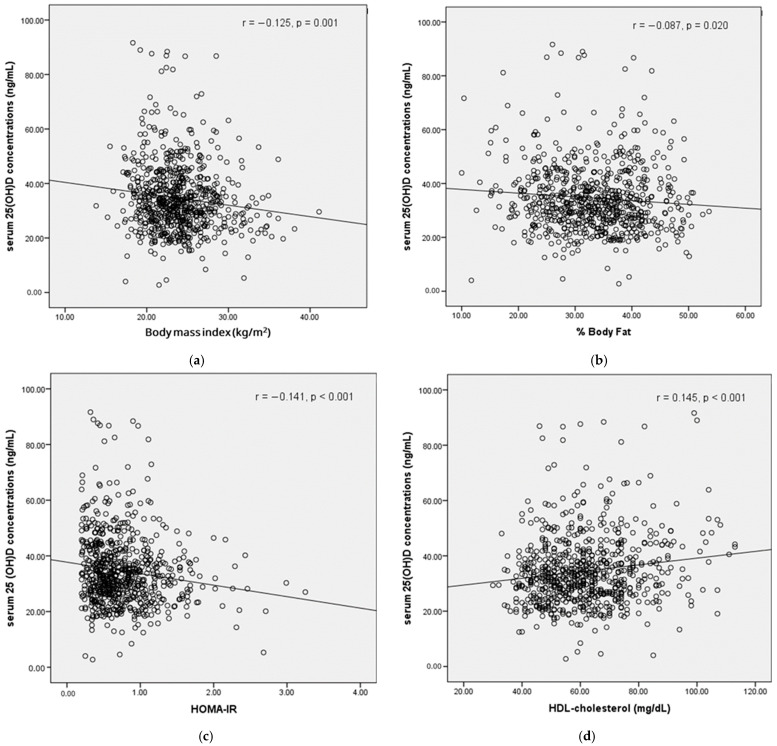
The relationship between serum 25(OH)D concentrations and (**a**) body mass index (BMI); (**b**) %body fat; (**c**) HOMA-IR; and (**d**) HDL cholesterol.

We further explored the relationship between vitamin D status and body composition. Serum 25(OH)D concentrations were negatively associated with weight (r = −0.129, *p* = 0.001), BMI (r = −0.125, *p* = 0.001), body fat mass (r = −0.128, *p* = 0.001), %body fat (r = −0.087, *p* = 0.020), visceral fat area (r = −0.101, *p* = 0.007), insulin (r = −0.141, *p* < 0.001), and HOMA-IR (r = −0.141, *p* < 0.001). In contrast, 25(OH)D levels were positively correlated with age (r = 0.100, *p* = 0.007) and HDL cholesterol (r = 0.145, *p* < 0.001) (Figure 1). However, no significant association was observed between vitamin D status and cognitive function, as measured by MMSE and MoCA scores.

The multivariate logistic regression analysis demonstrated that older age (adjusted OR = 1.07; 95% CI: 1.04–1.11, *p* < 0.001), higher BMI (adjusted OR = 1.07; 95% CI: 1.03–1.12, *p* = 0.002), and fewer years of education (adjusted OR = 0.88; 95% CI: 0.85–0.92, *p* < 0.001) were significant predictors of MCI after adjustment for HbA1C, HDL cholesterol, systolic blood pressure, and cardiovascular diseases (Table 4).

## 4. Discussion

Our study revealed a high prevalence of vitamin D inadequacy among elderly individuals in Thailand, despite it being a tropical country. We also identified a significant association between vitamin D status and adiposity, as well as a relationship between serum 25(OH)D levels and insulin resistance. However, no significant association was observed between vitamin D status and cognitive function, as measured by MMSE and MoCA scores, in participants with normal cognition or mild cognitive impairment.

The prevalence of vitamin D deficiency and vitamin D insufficiency among elderly individuals was high in the present study [11,45,46,47,48,49]. Even though sunlight exposure is abundant in Thailand, factors such as reduced cutaneous synthesis of vitamin D due to skin aging, lifestyle habits like sun avoidance, and urbanization likely contribute to low vitamin D levels [8,50]. Additionally, 33% of participants in our study had a BMI over 25 kg/m², indicating obesity within the Asian population, which is another risk factors for vitamin D deficiency [7,51,52]. Despite 28% of participants reporting the use of vitamin D supplementation, our study found that 40% of participants had 25(OH)D concentrations below 30 ng/mL, and 6.5% had levels below 20 ng/mL. This highlights that even with supplementation, achieving adequate vitamin D levels remains a challenge, particularly among older adults who have decreased cutaneous vitamin D synthesis.

The association between vitamin D deficiency and adiposity observed in our study aligns with previous reports showing an inverse relationship between serum 25(OH)D levels and BMI, body fat, and percentage of body fat [7,51]. This relationship can be explained by the sequestration of vitamin D in adipose tissue, which reduces its bioavailability, since adipose tissue acts as a reservoir for fat-soluble vitamins and higher adiposity may lead to lower circulating levels of 25(OH)D due to its storage in fat cells [53]. Additionally, vitamin D may play a role in modulating adiposity by inhibiting the differentiation of pre-adipocytes into mature adipocytes [54]. Our findings support the notion that higher body fat may reduce circulating vitamin D levels, which could be an important consideration for addressing vitamin D insufficiency in obese individuals.

We also found that serum 25(OH)D concentrations were inversely associated with insulin resistance, as measured by the HOMA-IR. This association could be due to several proposed mechanisms by which vitamin D improves insulin sensitivity. First, 1,25-dihydroxyvitamin D (1,25(OH)2D), the active form of vitamin D, influences insulin secretion and glucose metabolism by modulating calcium-flux-regulating receptors in the β-cell. Moreover, vitamin D suppresses the hyperactivity of the renin–angiotensin–aldosterone system (RAAS) and improves β-cell function [55,56]. Additionally, the inhibition of the RAAS by vitamin D may enhance insulin sensitivity in peripheral tissues such as skeletal muscle and adipose tissue, thereby improving glucose uptake and reducing insulin resistance. Furthermore, vitamin D may exert anti-inflammatory properties which play a role in mitigating chronic inflammation, which is a known contributor to insulin resistance [57]. This anti-inflammatory effect may improve insulin signaling pathways and reduce systemic insulin resistance. These mechanisms collectively highlight the potential role of vitamin D in regulating metabolic health, particularly in individuals with obesity or insulin resistance. However, further research is needed to elucidate the causal relationships and therapeutic implications of these findings.

Despite established links between vitamin D, adiposity, and insulin resistance, we found no significant association between serum 25(OH)D levels and cognitive function, as measured by the MMSE and MoCA scales, in participants with normal cognitive function and MCI. Our findings were consistent with several studies [28,29,32,33,35,36], which also found no evidence of an association between vitamin D status and cognitive impairment. For instance, a large long-term cohort study in elderly men conducted in Sweden revealed no association between baseline vitamin D status and the long-term risk of cognitive impairment or dementia over an 18-year follow-up period (median follow-up = 12 years) [29]. Similarly, a prospective study in Switzerland found no evidence of a relationship between vitamin D status and cognitive impairment or dementia during a 2-year follow-up in very elderly patients [36]. In line with these findings, the Canadian Study of Health and Aging also demonstrated no significant association between vitamin D status and cognitive decline, dementia, or Alzheimer’s disease (AD) during a mean follow-up of 5.4 years [28]. In contrast, others studied have suggested potential association between low vitamin D levels and an increased risk of Alzheimer’s disease and other forms of dementia [24,25,26,27,30,31,34]. A prospective study showed that vitamin D deficiency was associated with an increased risk of all-cause dementia and AD, during a median follow-up of 5.6 years [25]. However, a substantial number of subjects were excluded due to missing information of dementia status and lack of data on vitamin D status [25]. Similarly, other longitudinal studies demonstrated a significant association between baseline vitamin D status and cognitive decline and dementia over a follow-up period of 6 to 13 years [26,27,30]. The finding from the Third National Health and Nutrition Examination Survey (NHANES III) suggested that vitamin D deficiency was linked to an increased risk of cognitive impairment in elderly [31]. Interestingly, a previous analysis of NHANES III did not show an association between vitamin D status and cognitive function [32] which may be attributed to differences in the cognitive assessments used, such as raw scores versus composite scores.

Overall, the discrepancies in these findings may be attributed to the characteristics and age of the study participants, differences in vitamin D measurement methods [24,25,27,29,36], baseline vitamin D status, variations in the cognitive assessment tools used to define cognitive impairment [24,25,26,35] across studies, and the number of participants who had cognitive impairment, AD, and other dementias. The most significant factor may be the potential impact of vitamin D supplementation, which could have introduced bias and influenced the outcomes of our study. Previous studies have suggested a U-shaped association between vitamin D status and cognitive impairment, where both low and high levels of vitamin D are associated with poorer cognitive outcomes [58]. However, this association was not observed in individuals who were not using vitamin D supplementation. In our study, we conducted subgroup analyses comparing participants with and without vitamin D supplementation and found consistent results, indicating no significant association between vitamin D status and cognitive function in either group. While vitamin D receptors are widely expressed in the brain, and vitamin D has been shown to exert neuroprotective effects in animal models [21], human studies on the relationship between vitamin D and cognitive function have produced mixed results [23,46,47,48,49].

The lack of a significant association between vitamin D status and cognitive function in our study may be attributed to several factors. First, we did not include individuals with advanced dementia. Second, our study utilized MMSE and MoCA tests to assess cognitive functions, which have limitations as screening tests with narrow score ranges, making it more difficult to differentiate cognitive functions between MCI patients and normal individuals compared to other comprehensive neurological tests. Third, up to 28% of participants were taking vitamin D supplementation, which could have influenced vitamin D levels and potentially masked any underlying relationship with cognitive function. Unfortunately, we were unable to determine the specific effects of vitamin D supplementation on cognitive outcomes due to the lack of detailed dosage and duration data. It is possible that vitamin D deficiency alone may not be sufficient to drive cognitive decline, but rather it acts in conjunction with other risk factors such as age, genetic predisposition, vascular health, and metabolic dysfunction. These findings highlight the complex pathophysiology of cognitive decline, which involves multiple factors beyond vitamin D status.

We acknowledge several potential limitations in our study. First, we used only baseline data from the prospective cohort study; therefore, we could not establish causality between vitamin D status, cognitive function, and body composition. Longitudinal data from our cohort, with follow-up measurements, will provide more insight into the temporal relationships between these variables. Second, our study did not include patients with advanced dementia, such as those with Alzheimer’s disease. Additionally, the limited number of participants with MCI may have reduced the statistical power to detect a potential association between vitamin D status and cognitive function. Third, we lacked information on sun exposure, vitamin D supplement dosages, duration, and their forms. Additionally, dietary vitamin D data in the Thailand food database were unavailable. Lastly, data on alcohol use, smoking, and vitamin D supplementation were self-reported, which may have introduced potential recall bias.

Despite these limitations, our study is significant, and we emphasized the high prevalence of vitamin D inadequacy and its association with adiposity and insulin resistance. These findings underscore the need for public health strategies aimed at improving vitamin D status among older adults in Thailand. Encouraging outdoor activity, particularly among the elderly, to increase sun exposure may be one of the most accessible and effective ways to enhance endogenous cutaneous vitamin D synthesis. Given the potential role of vitamin D in modulating metabolic and inflammatory pathways, addressing vitamin D deficiency may have broader implications for preventing chronic diseases such as type 2 diabetes and cardiovascular disease, in addition to cognitive decline. Nevertheless, our results suggested that vitamin D status may not be a direct determinant of mild cognitive impairment in this population. Further research, particularly studies with longer follow-up periods and inclusion of patients with dementia with details about vitamin D supplementation, is needed to clarify the potential role of vitamin D in cognitive decline and neurodegenerative diseases.

## 5. Conclusions

Our study highlights the high prevalence of vitamin D inadequacy among elderly individuals in Thailand and its inverse association with adiposity and insulin resistance. However, we did not find a significant association between vitamin D status and cognitive function in participants with normal cognition and MCI. The findings do not support a direct role of vitamin D status in cognitive decline in this population. Further research, particularly studies with longer follow-up periods and inclusion of patients with more advanced cognitive impairment, is needed to clarify the potential role of vitamin D in cognitive decline and neurodegenerative diseases. These findings emphasize the importance of addressing vitamin D deficiency in the elderly population to enhance overall health outcomes.

## Figures and Tables

**Table 1:** Clinical characteristics, comorbid diseases, and cognitive score of study participants.

Variables	Total	MCI	Normal	MD (95%CI)	*p*-Value
	*n* = 718	*n* = 248	*n* = 470		
Age, years	65.7 (5.8)	67.8 (6.0)	64.6 (5.4)	−3.12 (−3.98, −2.25)	<0.001
Age groups ^‡^, *n* (%)					
≥65 years	403 (56.1)	172 (69.4)	231 (49.1)	2.34 (1.69, 3.24)	<0.001
<65 years	315 (43.9)	76 (30.6)	239 (50.9)	1	
Male ^‡^, *n* (%)	202 (28.1)	77 (31.0)	125 (26.6)	1.24 (0.89, 1.74)	0.207
Education, years	14.3 (4.7)	12.3 (5.2)	15.4 (4.0)	3.08 (2.40, 3.77)	<0.001
Education, years ^‡^, *n* (%)					
≤6	62 (8.6)	38 (15.3)	24 (5.1)	3.36 (1.97, 5.75)	<0.001
>6	656 (91.4)	210 (84.7)	446 (94.9)	1	
Systolic blood pressure, mmHg	131.7 (17.8)	134.1 (18.7)	130.3 (17.2)	−3.78 (−6.64, −0.92)	0.010
Diastolic blood pressure, mmHg	75.6 (11.0)	76.4 (9.7)	75.2 (11.5)	−1.30 (−3.05, 0.47)	0.150
Alcohol consumption ^‡^, *n* (%)	241 (33.6)	80 (32.3)	161 (34.3)	0.91 (0.66, 1.27)	0.590
Smoking ^‡^, *n* (%)	31 (4.3)	8 (3.2)	23 (4.9)	0.65 (0.29, 1.47)	0.296
***Comorbid diseases***					
Type 2 diabetes mellitus ^‡^, *n* (%)	43 (6.0)	16 (6.5)	27 (5.7)	1.13 (0.60, 2.14)	0.704
Hypertension ^‡^, *n* (%)	200 (27.9)	77 (31.0)	123 (26.2)	1.27 (0.91, 1.78)	0.166
Dyslipidemia ^‡^, *n* (%)	265 (36.9)	99 (39.9)	166 (35.3)	1.22 (0.89, 1.67)	0.225
Cardiovascular disease ^‡^, *n* (%)	22 (3.1)	12 (4.8)	10 (2.1)	2.34 (1.00, 5.49)	0.045
Vitamin D supplementation ^‡^, *n* (%)	199 (27.7)	62 (25.0)	137 (29.1)	0.81 (0.57, 1.15)	0.238
***Cognitive tests***					
MMSE scores	27.3 (2.0)	26.1 (2.3)	27.9 (1.5)	1.83 (1.55, 2.10)	<0.001
MoCA scores	25.2 (3.2)	21.8 (2.4)	27.1 (1.6)	5.29 (5.00, 5.58)	<0.001

Data are presented as mean (standard deviation) and a number with percentage (%). MCI, mild cognitive impairment; MMSE, Mini-Mental State Examination; MoCA, The Montreal Cognitive Assessment; ‡, odds ratio; MD, mean difference; CI, confidence interval.

**Table 2:** Body composition of study participants.

Variables	Total	MCI	Normal	MD (95%CI)	*p*-Value
	*n* = 718	*n* = 248	*n* = 470		
Weight, kg	60.6 (11.6)	61.7 (12.1)	60.1 (11.2)	−1.59 (−3.37, 0.20)	0.082
BMI, kg/m^2^	23.9 (3.7)	24.6 (4.2)	23.5 (3.4)	−1.12 (−1.69, −0.54)	<0.001
BMI groups ^‡^, *n* (%)					
<18.5 kg/m^2^	33 (4.6)	9 (3.7)	24 (5.1)	1	0.001
18.5–22.9 kg/m^2^	272 (38.2)	88 (35.9)	184 (39.4)	1.27 (0.57, 2.85)	
23.0–24.9 kg/m^2^	172 (24.2)	52 (21.2)	120 (25.7)	1.12 (0.50, 2.66)	
25.0–29.9 kg/m^2^	186 (26.1)	66 (26.9)	120 (25.7)	1.47 (0.64, 3.34)	
≥30.0 kg/m^2^	49 (6.9)	30 (12.2)	19 (4.1)	4.21 (1.62, 10.97)	
BMI groups ^‡^, *n* (%)					
<25.0 kg/m^2^	477 (67.0)	149 (60.8)	328 (70.2)	1	0.011
≥25.0 kg/m^2^	235 (33.0)	96 (39.2)	139 (29.8)	1.52 (1.10, 2.1)	
Waist circumference, cm	81.9 (9.4)	83.2 (10.4)	81.3 (8.8)	−1.90 (−3.36, −0.45)	0.011
Hip circumference, cm	93.6 (6.1)	94.4 (6.5)	93.2 (5.8)	−1.15 (−2.09, −0.21)	0.017
Waist–hip Ratio	0.87 (0.05)	0.87 (0.06)	0.86 (0.5)	−0.01 (−0.02, −0.003)	0.041
Body fat, kg	20.3 (7.0)	21.5 (7.8)	19.7 (6.6)	−1.74 (−2.82, −0.65)	0.002
% Body fat	33.2 (7.9)	34.3 (8.3)	32.6 (7.6)	−1.74 (−2.96, −0.51)	0.005
Visceral fat area, cm^2^	101.6 (40.7)	108.7 (44.3)	97.8 (38.2)	−10.92 (−17.18, −4.66)	<0.001
Skeletal muscle mass, kg	21.6 (4.9)	21.5 (4.9)	21.7 (5.0)	0.12 (−0.64, 0.89)	0.750
% Skeletal muscle mass	35.7 (4.7)	35.1 (4.8)	36.1 (4.5)	0.99 (0.28, 1.71)	0.007

Data are presented as mean (standard deviation) and a number with percentage (%). MCI, mild cognitive impairment; BMI, body mass index; ‡, odds ratio; MD, mean difference; CI, confidence interval.

**Table 3:** Biochemical data of study participants.

Variables	Total	MCI	Normal	MD (95%CI)	*p*-Value
	*n* = 718	*n* = 248	*n* = 470		
***Biochemical parameters***					
Total 25(OH)D levels, ng/mL	34.5 (12.6)	34.1 (12.0)	34.7 (12.9)	0.69 (−1.25, 2.62)	0.488
25(OH)D_2_, ng/mL	8.4 (15.5)	7.4 (14.6)	9.0 (15.9)	1.60 (−0.79, 3.98)	0.189
25(OH)D_3_, ng/mL	24.9 (10.6)	25.4 (10.8)	24.6 (10.5)	−0.88 (−2.52, 0.76)	0.290
Vitamin D status					
Vitamin D deficiency ^‡^ (< 20 ng/mL)	47 (6.5)	15 (6.1)	32 (6.8)	0.88 (0.47, 1.66)	0.695
Vitamin D insufficiency ^‡^ (20–30 ng/mL)	240 (33.4)	86 (34.7)	154 (32.8)	1.09 (0.79, 1.54)	0.606
Vitamin D inadequacy ^‡^ (< 30 ng/mL)	287 (40.0)	101 (40.7)	186 (39.6)	1.05 (0.77, 1.44)	0.765
Insulin, µIU/mL	5.8 (3.4)	6.1 (3.8)	5.6 (3.1)	−0.41 (−0.93, 0.11)	0.122
HOMA-IR	0.77 (0.43)	0.81 (0.49)	0.74 (0.40)	−0.06 (−0.13, 0.003)	0.062
Cholesterol, mg/dL	208.6 (42.7)	204.3 (42.0)	210.9 (43.0)	6.64 (0.06, 13.22)	0.048
TG, mg/dL	106.9 (52.4)	107.3 (49.7)	106.7 (53.9)	−0.65 (−8.74, 7.45)	0.876
LDL-C, mg/dL	132.8 (40.7)	129.1 (39.5)	134.7 (41.1)	5.60 (−0.66, 11.87)	0.080
HDL-C, mg/dL	61.8 (15.0)	60.3 (15.2)	62.6 (14.9)	2.32 (0.01, 4.64)	0.049
Fasting plasma glucose, mg/dL	91.2 (11.7)	92.5 (12.7)	90.6 (11.0)	−1.96 (−3.75, −0.16)	0.033
HbA1C, %	5.7 (0.4)	5.7 (0.5)	5.6 (0.4)	−0.12 (−0.18, −0.05)	<0.001
HbA1C group ^‡^, *n* (%)					
<5.7%	386 (54.0)	115 (46.6)	271 (57.9)	1	0.004
≥5.7%	329 (46.0)	132 (53.4)	197 (42.1)	1.58 (1.16, 2.15)	

Data are presented as mean (standard deviation) and a number with percentage (%). MCI, mild cognitive impairment; HOMA-IR, homeostasis model assessment of insulin resistance; TG, Triglyceride; LDL-C, low-density lipoprotein cholesterol; HDL-C, high-density lipoprotein cholesterol; ng/mL; ‡, odds ratio; MD, mean difference; CI, confidence interval.

**Table 4:** A univariate and multivariate logistic regression of factors associated with mild cognitive impairment.

Variables	Univariate Analysis		Multivariate Analysis	
	Crude OR (95% CI)	*p*-Value	Adjusted OR (95% CI)	*p*-Value
Total 25(OH)D levels, ng/mL	0.996 (0.983, 1.008)	0.487		
Age, years	1.102 (1.071, 1.133)	<0.001	1.073 (1.041, 1.106)	<0.001
Education, years	0.864 (0.833, 0.896)	<0.001	0.882 (0.849, 0.916)	<0.001
Sex, male	1.243 (0.886, 1.743)	0.207		
Systolic blood pressure, mmHg	1.012 (1.003, 1.021)	0.010		
BMI, kg/m^2^	1.082 (1.038, 1.128)	<0.001	1.072 (1.025, 1.121)	0.002
HDL-C, mg/dL	0.990 (0.979, 1.000)	0.050		
Cardiovascular disease	2.339 (0.996, 5.493)	0.051		
HbA1C, mg/dL	1.809 (1.269, 2.578)	0.001		

OR, odds ratio; CI, confidence interval; BMI, body mass index; HDL-C, high-density lipoprotein cholesterol.

## Data Availability

The data presented in the current study are not publicly available owing to privacy and ethical restrictions. However, data are available from the corresponding authors upon reasonable request.
